# Supplementary material for: Nasal Symptoms in Asthmatic Patients under Treatment with Anti-IL-5 Monoclonal Antibodies. A Real-Life Cohort Study
Source: J Clin Med. 2022 Nov 29;11(23):7056. doi: 10.3390/jcm11237056 (PMC9740481; doi:10.3390/jcm11237056)
Supplement: Supplementary file 1 [file jcm-11-07056-s001.zip › jcm-2016971-supplementary.pdf]

Supplementary File S1: distribution of the risk factors and comorbid conditions

Comparison between groups	Total (n=38)	Benralizumab (n=17)	Mepolizumab (n=19)	Reslizumab (n=2)	Statistical analysis
**Emphysema**	0 (0%)	0 (0%)	0 (0%)	0 (0%)	NA
**Bronchiectasis**	5 (13.16%)	1 (5.88%)	4 (21.05%)	0 (0%)	chi2(2) = 2.1269<br>Pr = 0.345
**Apnea**	11 (28.95%)	6 (35.29%)	5 (26.32%)	0 (0%)	chi2(2) = 1.2117<br>Pr = 0.546
**Asthma**	38 (100%)	17 (100%)	19 (100%)	2 (100%)	NA
**Reflux**	16 (42.11%)	10 (58.82%)	5 (26.32%)	1 (50.0%)	chi2(2) = 3.9435<br>Pr = 0.139
**Diabetes**	2 (5.26%)	2 (11.76%)	0 (0%)	0 (0%)	chi2(2) = 2.6078<br>Pr = 0.271
**Hypercholesterolemia**	7 (18.42%)	5 (29.41%)	2 (10.53%)	0 (0%)	chi2(2) = 2.6061<br>Pr = 0.272
**Hypertension**	6 (15.79%)	3 (17.65%)	3 (15.79%)	0 (0%)	chi2(2) = 0.4191<br>Pr = 0.811
**Alcohol consumption**	4 (10.53%)	2 (11.76%)	2 (10.53%)	0 (0%)	chi2(2) = 0.6417<br>Pr = 0.726
**Former Smoking habit**	11 (28.95%)	5 (29.41%)	6 (31.58%)	0 (0%)	chi2(4) = 2.3182<br>Pr = 0.677

N-ERD	8 ()	5 ()	2 ()	1 ()	chi2(2) = 2.9897<br>Pr = 0.224
